# Identification of apolipoprotein using feature selection technique

**DOI:** 10.1038/srep30441

**Published:** 2016-07-22

**Authors:** Hua Tang, Ping Zou, Chunmei Zhang, Rong Chen, Wei Chen, Hao Lin

**Affiliations:** 1 Department of Pathophysiology, Southwest Medical University, Luzhou 646000, China; 2 Key Laboratory for NeuroInformation of Ministry of Education, School of Life Science and Technology and Center for Informational Biology, University of Electronic Science and Technology of China, Chengdu 610054, China; 3 Department of Physics, School of Sciences, Center for Genomics and Computational Biology, North China University of Science and Technology, Tangshan 063009 China

## Abstract

Apolipoprotein is a kind of protein which can transport the lipids through the lymphatic and circulatory systems. The abnormal expression level of apolipoprotein always causes angiocardiopathy. Thus, correct recognition of apolipoprotein from proteomic data is very crucial to the comprehension of cardiovascular system and drug design. This study is to develop a computational model to predict apolipoproteins. In the model, the apolipoproteins and non-apolipoproteins were collected to form benchmark dataset. On the basis of the dataset, we extracted the *g*-gap dipeptide composition information from residue sequences to formulate protein samples. To exclude redundant information or noise, the analysis of various (ANOVA)-based feature selection technique was proposed to find out the best feature subset. The support vector machine (SVM) was selected as discrimination algorithm. Results show that 96.2% of sensitivity and 99.3% of specificity were achieved in five-fold cross-validation. These findings open new perspectives to improve apolipoproteins prediction by considering the specific dipeptides. We expect that these findings will help to improve drug development in anti-angiocardiopathy disease.

Apolipoproteins (Apo) are proteins that bind lipids to form lipoproteins, whose main function is to transport the lipids through the lymphatic and circulatory systems^1^. There are two major types of apolipoproteins. One type has mostly beta-sheet structure and associates with lipid droplets irreversibly. They can form low-density lipoprotein. The other type consists of alpha-helices and associates with lipid droplets reversibly. Most proteins of the second type can form high-density lipoprotein particles. Several studies have demonstrated that the apolipoproteins have important functions in cardiovascular system, digestive system, and etc. For example, ApoE mediates the transport and uptake of cholesterol and lipid by way of its high affinity interaction with different cellular receptors^2^. ApoA1 is thought to act primarily in intestinal lipid absorption^1^. Thus, accurate identification of the apolipoprotein is very crucial to the comprehension of cardiovascular and digestive system as well as drug design.

The available of huge amounts of proteins generated in postgenomic age provides us an opportunity to design computational methods to timely and precisely predict protein functions. In fact, in the past two decades, a great deal of works have been focused on protein structure and function prediction by using machine learning methods, such as support vector machine (SVM)^3^,^4^,^5^, random forest (RF)^6^, Increment of diversity (ID)^7^, the Mahalanobis discriminant^8^, ensemble classifiers^9^,^10^, feature selection techniques^11^, etc. Amino acid composition^12^ was always selected as the key feature to formulate protein sequence. However, the residue-order information was completely lost. To improve the description about protein samples, the *n*-peptide information^13^, N-terminal amino acid sequence^14^, secondary structure involved features^15^,^16^ were proposed. Evolution information generated by PSI-BLAST was also considered by several studies^17^,^18^,^19^,^20^. To incorporate the physicochemical properties with residue-order information, the pseudo amino acid composition (PseAAC) was developed to efficiently improve prediction quantity^21^,^22^,^23^.

Based on these methods, many protein prediction issues such as protein subcellular localization^22^, protein structural classes^24^,^25^, enzyme classification^26^,^27^ have been studied. However, to the best of our knowledge, no computational method was proposed to predict apolipoproteins. The appearance of a great number of protein data provides us an opportunity to statistically study and predict apolipoproteins. Thus, this study aims to develop a computational method to identify apolipoproteins. High quality dataset was constructed to train and test the proposed method. Informative features were optimized by using feature selection technique and then inputted into SVM for performing prediction. Finally, based on the optimal model, we established a webserver called **ApoliPred** which can be freely used by all scholars. The following section will introduce these steps in detailed.

## Results

### Prediction Performance

The predictive performances of *g*-gap dipeptide composition were investigated by using SVM with five-fold cross-validation test. To investigate whether a specific type of *g*-gap dipeptides is mostly contributable for apolipoproteins identification, we varied the interval residue parameter *g* from 0 to 9. After checking the overall accuracies (*OA*s) obtained by different *g*-gap dipeptide composition, we found that the *OA* of 3-gap dipeptide composition is 94.2% in five-fold cross-validation which is higher than that of other *g*-gap dipeptide compositions.

**Figure 1:**
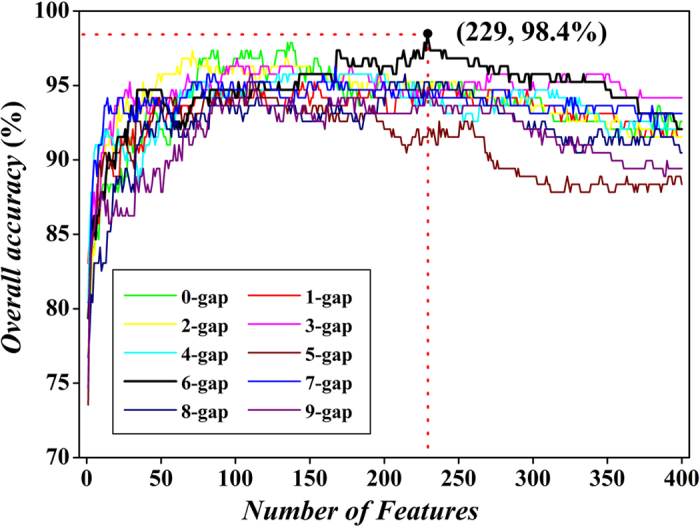
A plot showing the IFS procedure for discriminating apolipoproteins from non-apolipoproteins. When the top 229 6-gap dipeptides were used to perform prediction, the overall success rate reaches an IFS peak of 98.4% in five-fold cross-validation.

The above results implied that the information of apolipoproteins mainly stores in the correlation of two residues with 3 residues interval. However, the noises or redundant information maybe result in the poor predictive capabilities of other *g*-gap dipeptide compositions. Thus, feature selection method *F*-score as defined in Eq. 3 was used to exclude noise and redundant information. For an arbitrary *g*-gap dipeptide composition, it has 400 features. On the basis of Eq. 3, a total of 400 *F*-scores were calculated for the 400 features. Subsequently, the 400 features were ranked according to their *F*-scores. The incremental feature selection (IFS) is used to determine the optimal number of features according to the following steps. Firstly, the feature with the maximum *F*-score was selected as the input of SVM. The *OA* was calculated to evaluate the performance of this feature. Secondly, the feature with the second maximum *F*-score was combined with the first feature to form a new feature subset. The *OA* was still used to estimate the performance of the new feature subset by using SVM. This process was repeated until 400 *OA*s were calculated. The best feature subset is the subset that can produce the maximum *OA*. By setting dimension of feature subset (the number of features) as abscissa and the *OA* as ordinate, we plotted 10 curves as shown in Fig. 1. From the figure, we noticed that the maximum *OA* of 98.4% can be achieved by 229 6-gap dipeptides which are regarded as the optimal feature subset. Thus, the final model was constructed by these features. The sensitivity (*Sn*) and specificity (*Sp*) are 96.2% and 99.3%, respectively. We also investigated the *OA* of optimal feature subset by using jackknife cross-validation. Results showed that *OA* is 97.35%, which also demonstrates that the optimal model is powerful.

By comparing the results of original and optimal *g*-gap dipeptide composition, we may draw a conclusion that feature selection not only improves the model’s performance, but also find the potential correlation between two residues. In fact, the above results show that, in apolipoprotein prediction, the correlation information mainly exists in 6-gap dipeptides. This demonstrates that the feature selection is a very valid way to discover the intrinsic characteristics of apolipoproteins.

### A heat map analysis

**Figure 2:**
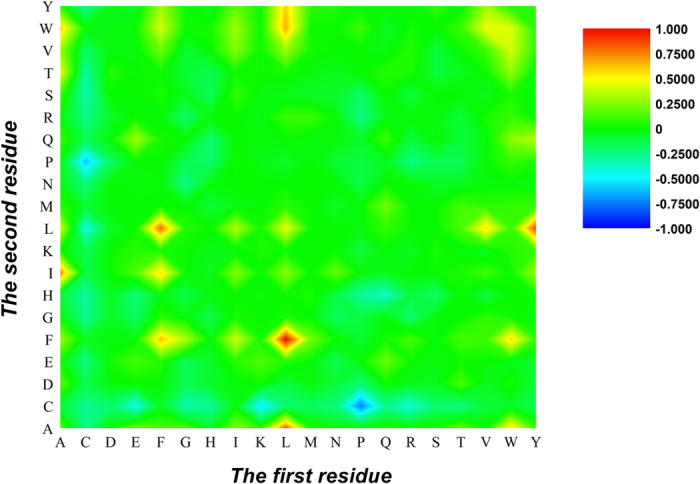
A heat map or chromaticity diagram for the *F*-scores of the 400 6-gap dipeptides. The blue boxes indicate that the features are enriched in apolipoproteins, while the red boxes indicate that the features are enriched in non-apolipoproteins.

To provide an overall and intuitive view for understanding the contribution of features, the preference of 6-gap dipeptide composition in both positive and negative datasets was investigated by using Eq. 4. If 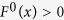, the *x*-th 6-gap dipeptide prefers apolipoprotein, otherwise it prefers non-apolipoprotein. Based on Eq. 4, a heat map was drawn in Fig. 2. The first and second residues of the 6-gap dipeptides were, respectively, listed in row and column of the heat map. Thus, each element in the heat map represents one of the 400 6-gap dipeptides. The color of the element was drawn according to its *F*-score. The features in red and blue boxes are positively and negatively correlated with apolipoproteins, respectively. It is obvious that the redder the element is, the more highly relevant with apolipoproteins it is, and vice versa. From the figure, we found that the residues Leu (L), Phe (F), Ile (I) and Trp (W) as well as their 6-gap correlations are abundant in apolipoproteins compare to non-apolipoproteins, whereas the residues Cys (C), Glu (E), K and P (blue) as well as their 6-gap correlations exhibit the opposite behavior. Thus, we further used 20 6-gap dipeptides with maximum *F*-scores to discriminate apolipoproteins from non-apolipoproteins. It shows that the overall accuracy reaches 91.0% in five-fold cross-validation. These results indicate that Leu (L), Phe (F), Ile (I), Trp (W), Cys (C), Glu (E), Lys (K) and Pro (P) (blue) are key features for apolipoprotein identification.

### Web-server guide

To provide the convenience for vast majority of scholars, a user-friendly web-server called **ApoliPred** was established. The server is freely access at http://lin.uestc.edu.cn/server/ApoliPred. A step-by-step guide on how to use the webserver is given below.

**Figure 3:**
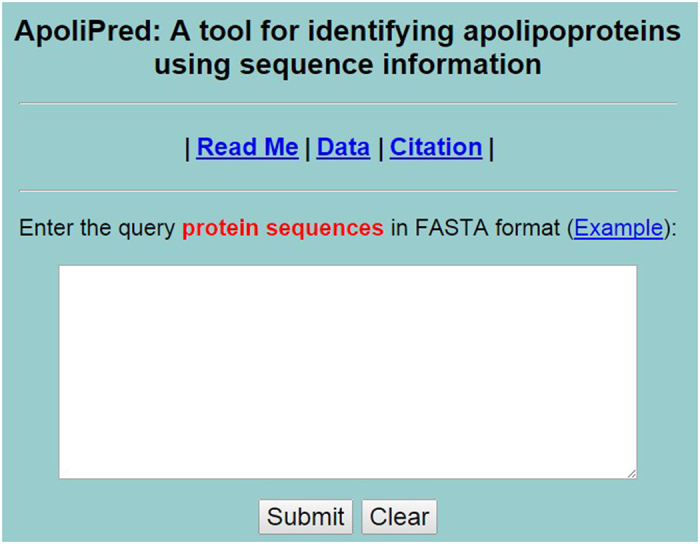
A semi-screenshot to show the top page of the **ApoliPred** webserver. Its website address is http://lin.uestc.edu.cn/server/apoliPred.

Users may open the web server at http://lin.uestc.edu.cn/server/ApoliPred and the top page of **ApoliPred** will appear on user’s computer screen, as shown in Fig. 3. The Read Me button provides a brief introduction about the predictor and the caveat when using it. The Data button provides the benchmark dataset used in this study to train and test the **ApoliPred** predictor. The Citation button given the relevant papers documenting the detailed development and algorithm of **ApoliPred**.

Users may type or copy/paste the query residue sequences into the input box at the center of Fig. 3. The input sequence must be in the FASTA format. By clicking on Example button, users can look at the example sequences in FASTA format. By clicking on the Submit button, the predicted results will appear.

## Discussion

The aim of this work is to build a predictive model to identify apolipoproteins. In fact, the programs such as BLAST and FASTA have been widely used in genomic and proteomic analysis or prediction based on similarity search. However, these programs are helplessness as facing low-similar sequences. Especially, more and more orphan genes were found. Few of them can be functional annotated in GenBank by similarity search. With the appearance of more and more orphan genes or low-similar proteins, it is urgent to develop a statistical predictive model. Thus, the study is very meaningful and important.

All results and models are derived from a strict and objective benchmark dataset. All data in benchmark dataset have been confirmed by biochemical experiments. Thus, the information extracted from such dataset is precise and reliable. Moreover, many papers have reported that the proposed model will be overestimated or bias if they are trained and tested by high similar sequences^22^,^28^. In this study, we have removed the redundant sequences by setting sequence identity to 40%. Thus, our models are reliable and harmonious.

Correlation of nucleotides or residues is the key carrier of genetic information. Therefore, we used *g*-gap dipeptide composition as features in prediction. However, the performances of models are far from satisfactory based on such fundamental information. To improve the accuracies and find out the real correlations hiding in protein sequences, a feature selection technique was applied to select optimal features. Results demonstrate that the technique can pick out informative features, dramatically improve the predictive performance and enhance the generalization abilities of the proposed models. Based on the correlation information and feature selection technique, our models did achieve promising results for recognition of apolipoproteins.

## Conclusion

In this study, a new tool, called **ApoliPred**, was established for accurate identification of potential novel apolipoproteins. In **ApoliPred**, a high-quality benchmark dataset was constructed by setting a series of standards, which can guarantee the reliable of the tool. Thus, the dataset has the potential to become a standard dataset in the development of computational methods in theoretical study of apolipoproteins. Moreover, a feature selection technique has been successfully applied to improve the performance. The special dipeptide distribution were discovered in apolipoproteins. Our results indicated that the proposed model can provide a high discriminative accuracy. We expect that these findings will help to improve drug development in anti-angiocardiopathy disease. The method proposed in this work can also be used in other field of bioinformatics and computational biology.

## Materials and Method

### Benchmark dataset

A strict and objective benchmark dataset can guarantee the reliable of the prediction model. Thus, all apolipoproteins were downloaded from the Universal Protein Resource (UniProt)^29^. For the purpose of obtaining a high quality data, the following steps were performed.

1. Choose the apolipoproteins which have been annotated in Swiss-Prot.
2. Exclude the proteins whose sequences contain illegal characters such as “B”, “J”, “O”, “U”, “X” and “Z”.
3. Select the proteins whose function (namely the Gene Ontology (GO)) have been annotated.
4. Remove homologous sequences by setting the cutoff threshold of CD-HIT to 0.4.

After following the above processes, we obtained a total of 53 apolipoproteins and 136 non-apolipoproteins which can be freely downloaded from http://lin.uestc.edu.cn/server/ApoliPred.

### Protein Feature Extraction

It is widely accepted that the functions of proteins correlate with their three-dimensional structures. Thus, we extracted features from the primary sequence of apolipoproteins and non-apolipoproteins. Dipeptide composition has been widely applied in protein classification and has achieved encouraging results^30^,^31^. However, it can only reflect the short-range correlation. In most cases, non-adjoining residues in primary sequence might be proximate in three-dimensional space. For example, in alpha helix and beta sheet, the hydrogen bonds are in charge of the connection of two non-adjoining residues. Thus, we used the *g*-gap dipeptide composition to describe the correlation of residues in protein primary sequence. Thus, a given protein **P** can be formulated by a 400-Dimension vector and defined as:


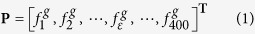


where **T** is the transposing operator, the 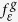 denotes the frequency of the *ε*-th type (*ε* = 1, 2, …, 400) of the *g*-gap dipeptide in protein **P** and can be calculated by:


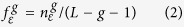


where the 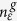 is the occurrence number of the *ε*-th type (*ε* = 1, 2, …, 400) of *g*-gap dipeptide in the protein **P**. The *L* in the denominator of Eq. 2 is the length of the protein **P**. If the parameter *g* is set to 0, the formulation degrades into the adjoining dipeptide composition which describes the correlation of two proximate residues. Thus, the *g*-gap dipeptide composition represents the direct correlation between two residues with *g* residues interval.

### Support vector machine (SVM)

Based on the statistical learning theory, Vapnik and his colleagues have developed a powerful and popular machine learning method called SVM. It is a supervised learning method which projects samples with low-dimension feature into a high-dimension Hilbert space and constructs a hyperplane to perform classification. Due to its good capability for non-linear classification and small sample classification, SVM has been widely applied in protein structure and function classification as well as DNA motif prediction^3^,^13^,^21^,^22^,^26^,^28^,^32^,^33^. Thus, we also used the SVM to perform the classification. A free software package LibSVM (version 3.2) was used to implement the SVM. The radial basis function (RBF) was chosen as the kernel function because it is more suitable for nonlinear classification than other kernel functions. To obtain the best performance, the grid search approach was applied to optimize regularization parameter *C* and the kernel width parameter *γ* by using 5-fold cross-validation.

### Feature selection technique

Generally, in statistical learning problem, not every feature has a positive contribution to the classification, especially for high dimension data. Some features are noise or redundant information which will reduce the predictive performance of classification models. Thus, it is very important to develop a method to evaluate the contribution of every feature to the classification. Based on this consideration, we proposed an *F*-score to describe the contribution of each feature.

Based on the hypothesis that if the sample variance of a feature between groups is larger than sample variance within groups^3^, the feature is suitable for classification, we defined the *F*-score of a feature *x* as follow.


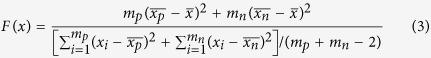


where 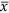, 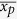 and 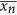 are the means of feature *x* in all samples, positive samples and negative samples, respectively. *m*_*p*_ and *m*_*n*_ are the number of samples in positive dataset or negative dataset, respectively. Thus, the numerator and denominator in Eq. 3 denote the variances between groups and within groups, respectively. It is obvious that the larger the *F*(x) is, the better capability the feature *x* has.

We used the following normalized function to scale the *F*(*x*) of the feature *x* as follow


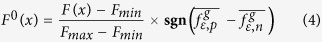


where *F*_min_ and *F*_max_ are the minimum and maximum *F* values of the 400 *g*-gap dipeptides. The 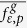 and 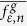 are the average frequencies of the *ε*-th *g*-gap dipeptide in positive sample dataset and negative sample set, respectively; **sgn** is the sign function. Thus, the upper limit and lower limit of 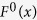 are 1 and −1, respectively.

### Performance evaluation

In statistical prediction, several test methods such as *n*-fold cross-validation test, jackknife cross-validation test, independent data test can be used to estimate the predictive performance of proposed method^34^. Jackknife cross-validation test is usually more suitable for small sample problem and always yields a unique results for a given benchmark dataset^28^,^35^,^36^, however, it is time-consuming. Thus, we used five-fold cross-validation in this study to evaluate the performance of our model.

To quantitatively evaluate the performance of models, the following three indexes called Sensitivity (*Sn*), Specificity (*Sp*) and Overall Accuracy (*OA*) were used and can be defined as:


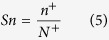



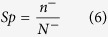



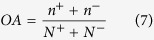


where *n*^+^ and *n*^−^ are the number of the correctly identified positive samples (i.e. apolipoproteins) and the number of the correctly identified negative samples (i.e. non-apolipoproteins), respectively; *N*^+^ and *N*^−^ are the number of the positive samples and the number of negative samples in the benchmark dataset, respectively.

## Additional Information

**How to cite this article**: Tang, H. *et al.* Identification of apolipoprotein using feature selection technique. *Sci. Rep.* **6**, 30441; doi: 10.1038/srep30441 (2016).
